# #Healthpromotion: A qualitative exploration of how dietitians can use social media to positively influence women aged 18–35 years

**DOI:** 10.1111/1747-0080.12765

**Published:** 2022-09-09

**Authors:** Danielle Shine, Michelle Minehan, Cathy Knight‐Agarwal

**Affiliations:** ^1^ Faculty of Health University of Canberra Bruce Australian Capital Territory Australia

**Keywords:** dietitians, health promotion, qualitative research, social media, young adult, women

## Abstract

**Aims:**

To understand how young adult women use social media, including which nutrition and health‐related content they prefer to view and why. Findings are intended to support dietitians to use social media more effectively for health promotion to reach, educate and positively influence young adult women.

**Methods:**

Qualitative research was conducted through semi‐structured interviews involving 10 women aged 18–35 years via Zoom videoconferencing. The interviews were recorded, transcribed verbatim and analysed using an interpretative phenomenological approach.

**Results:**

Young adult women use social media daily to view a wide variety of content, including nutrition and health‐related content. Three themes were identified: authenticity, engaging content, and affecting trust through selling products.

**Conclusion:**

To effectively use social media for health promotion, dietitians need to share their authentic voice while maintaining professional standards. Recommendations for effective social media engagement include using engaging content, infographics, and videos with closed captions. More research is needed to assess whether health promotion deployed via social media is effective at increasing nutrition knowledge, improving health literacy, and producing behaviour change.

## 1 INTRODUCTION

According to a report published in 2022,^1^ nearly 60% of the world's population are using internet‐based social networking applications (‘apps’) including Facebook, Instagram and TikTok to facilitate the creation of, and engagement with, user generated content. Collectively, these apps are commonly referred to as ‘social media’, a powerful medium that has significantly altered the way people generate, communicate and consume information.^2^, ^3^

From a public health perspective, social media presents novel, low‐cost opportunities to reach, educate, and positively influence many people, including young adult women (18–35 years) who represent one of the largest, most engaged social media populations in the world.^1^ Although research indicates that Facebook can be used to bolster health promotion targeting improved health literacy and dietary behaviours among women >18 years,^4^, ^5^, ^6^ less is known about the feasibility of Instagram and TikTok. This is concerning, given both apps continue to grow in popularity among young adult women^7^, ^8^ and despite increasing scrutiny about their inadvertent cultivation of dangerous diet cultures.^9^, ^10^, ^11^ Moreover, a growing number of unqualified, non‐health professional, social media ‘influencers’ propagate erroneous nutrition and health‐related information via this medium.^12^, ^13^, ^14^, ^15^ Although Instagram and TikTok have made efforts to curtail COVID‐19 and vaccine misinformation,^16^, ^17^ nothing has been done to attenuate nutrition misinformation. Consequently, the health and well‐being of social media users, including young adult women, is at high risk.^18^, ^19^

A more credible and influential nutrition presence on social media is warranted to support mitigation of nutrition and health‐related misinformation. Accredited Practising Dietitians are credentialled nutrition experts who provide credible, evidence‐based information that can support people to improve their food and health literacy.^20^ Research indicates that one of the main barriers to social media use among dietitians is a lack of knowledge about how to use it effectively for health promotion.^21^ Moreover, a review of the literature uncovered a dearth of research exploring the natural social media practices and preferences of women between 18–35 years. This intelligence is crucial to the development of social media content that reaches, resonates, thus, is utilised by this population. Therefore, the aim of this study was to explore the type/s of social media content, including nutrition and health‐related content, that young adult women (18–35 years) prefer to view and why.

## 2 METHODS

Qualitative research is used to gain an understanding of an individual's underlying motivations, actions and thoughts.^22^ The researchers consider reality to be socially constructed, thus, aim to produce subjective findings through a process of inductive reasoning. Instead of generating a series of hypotheses, the research presented here has been guided by a semi‐structured interview guide that focuses on the examination of experience which suggests a phenomenological course of inquiry is appropriate. Interpretative phenomenological analysis (IPA) is rooted in phenomenology, that is, it is concerned with individuals' lived experience and how they make sense of that experience.^23^

This paper is reported according to the Standards for Reporting Qualitative Research.^24^ Following ethical approval from the University of Canberra Human Research Ethics Committee (UC HREC‐9195), women were purposively recruited via advertisements displayed on campus noticeboards and social media apps including Facebook and Instagram. Purposive sampling requires that individuals are deliberately selected with an explicit purpose in mind, namely, to address the research aim and because they are rich sources of data in relation to this.^25^ To be eligible for inclusion, women were required to be English speaking, aged between 18 and 35 years, and users of at least one social media app, for example, Facebook, Instagram or TikTok. Women could be internationally located, however, they had to have access to a computer (or smartphone) with consistent internet connection and Zoom videoconferencing.^26^ Participants provided signed informed consent prior to interviews.

Ten women volunteered to take part in the project, with an average age of 27.2 years (Table 1). Just over half of the women recruited were tertiary educated (*n* = 6). One‐to‐one interviews were conducted via Zoom (Zoom Video Communications Inc.), between the primary researcher and each participant, over 8 weeks during June and July 2021. Participants elected interview times that were convenient for them. Interviews ranged from approximately 60–100 min in length. A semi‐structured interview guide was developed based upon a review of the published literature (see Online Supplementary Material S1). This was piloted before the main interviews took place to allow the primary researcher to test the interview questions and gain practice in interviewing. The majority of questions were kept deliberately open, allowing participants to talk at length, without judgement. A faciliatory interview style that included the use of verbal and non‐verbal cues was employed. An iterative approach was used to allow unanticipated lines of conversation to be explored. Each interview was audio recorded and transcribed verbatim, then entered into a word processing document for analysis. In total, 10 interviews were conducted.

**TABLE 1:** Participant demographical information for ten women who participated in the study

Participants	Age	Country of residence	Ethnicity	Social media app/s used most frequently	Years of social media use	Education level
Student dietitian	23	Australia	Italian	Facebook, Instagram, TikTok	11	Tertiary educated
Entertainer (actor)	18	United Kingdom	Asian	Facebook, Instagram, TikTok	7	Non‐tertiary educated
Artist	26	United States of America	Hispanic	Instagram	10	Non‐tertiary educated
Marketing/Communication specialist. Social media expertise.	29	United Kingdom	Caucasian	Facebook, Instagram, TikTok	15	Tertiary educated
Public health nutritionist	34	Australia	Caucasian	Facebook, Instagram	15	Tertiary educated
Student dietitian	21	Australia	Asian	Facebook, Instagram, YouTube	11	Tertiary educated
Marketing/Communication specialist. Social media expertise.	31	United States of America	Caucasian	Facebook, Instagram, Twitter	15	Non‐tertiary educated
Stay at home mum	30	United Kingdom	African American	Facebook, Instagram, TikTok	10	Non‐tertiary educated
Business development executive	31	Australia	Caucasian	Facebook, Instagram	14	Tertiary educated
Marketing/Communication specialist. Social media expertise.	29	United States of America	Caucasian	Facebook, Instagram	15	Tertiary educated

Data analysis was guided by Smith et al.'s IPA protocol.^23^ Step 1 involved full data immersion via reading/re‐reading the transcript and listening/re‐listening to the audio recording which increased opportunities for new insights to be made. Distinctive phrases and emotional responses were highlighted. The primary researcher recorded observations and reflections, including comments associated with personal reflexivity, given this can affect participants' responses. Step 2 involved initial note taking, with attention paid to descriptive, linguistic and conceptual comments made by the participant. Notes were transformed into emerging themes via concise phrases, with data remaining grounded in the particularity of the participants' responses. Step 3 involved developing and clustering concise statements (emergent themes) to capture and reflect understanding. Initially, these were chronologically ordered, however, re‐ordering took place to support identification of connections between identified themes. Step 4 introduced structure. Clusters of themes identified during Step 3 were named, grouped and tabulated to support clear depiction of the relationship between themes. Each participant's data set was approached in isolation to the other, to allow identification of new themes, prior to searching for patterns across all participant data sets. Once completed, a final cross analysis was conducted to support identification of commonalities and differences across all participant responses.

Ensuring trustworthiness throughout IPA is crucial to ensuring research acceptability and usefulness.^27^, ^28^ According to Guba,^29^ four criteria, namely, credibility, transferability, dependability and confirmability can be employed to ensure research validity, generalisability, reliability and objectivity. To optimise credibility, participants were assured anonymity and the freedom to withdraw from the research at any time, without prejudice. The primary researcher used reflective journaling to identify perspective and personal biases. Data accuracy, collection and storage was assured via dependability and confirmability audits. Specifically, a supervisory panel regularly reviewed the primary researcher's field notes and reflective journaling against participant transcripts to ensure conclusions made were internally coherent. Data coding was regularly compared and reviewed among a supervisory panel. Member checking was used to test analysis with participants.

## 3 RESULTS

Data analysis revealed three themes: authentic people are the most credible social media influencers; social media content must be engaging and easy to understand; selling on social media may dilute authenticity, diminish trust and turn participants away from social media content.

Theme 1: Authentic people are the most credible social media influencers. Specifically, participants preferred to follow ‘genuine’, ‘sincere’, ‘raw’, and ‘real’ people who were willing to show their ‘true’ self, as opposed to their best self via social media. According to participants, authentic people on social media were those who shared unedited photos and/or videos of themselves whereby their real skin tones and textures, as well as body shapes and size, can be seen. Similarly, authentic people were those who share openly about real‐life experiences, both positive and negative. In this regard, the stripping away of pretence was paramount to authenticity for participants in this study. Namely, they were deterred by people who appeared too ‘polished’ or rehearsed, living an idyllic, unblemished existence.

*I'm so sceptical of anyone who constantly shares perfect, heavily filtered images or videos of themselves in yoga poses, or making food you never actually see them eat… Also equally annoying are those people who share overly rehearsed videos, y'know, the ones where they dance to some random song while pointing to words on the screen to share information? Ugh, it's so cringy and inauthentic, I'm like: stop trying so hard!* (Participant 1)

All participants accessed nutrition and health‐related information on social media. Most preferred this content to be created by tertiary educated health professionals including Accredited Practising Dietitians, given they are appropriately qualified to disseminate scientific information and provide expert advice. Conversely, participants expressed their disdain for non‐health professionals including ‘holistic health coaches’, ‘biohackers’, personal trainers, and non‐tertiary educated nutritionists who routinely shared inaccurate nutrition and health‐related content on social media, thus, contributed to the spread of misinformation. In addition, several participants voiced their frustration about social media echo chambers perpetuated by inauthentic people who routinely shared nutrition and health‐related content based on faulty logic. This includes people who encouraged others to adopt restrictive diets, consume specific ingredients to ‘cleanse toxins’ from their body, and/or forgo important medical treatments including evidence‐based cancer therapies and COVID‐19 vaccinations.

Despite a preference for credible social media content created by tertiary educated health professionals, participants followed a higher number of non‐health professionals on social media. This indicates that while tertiary qualifications mattered when accessing nutrition and health‐related information, overall, the personal attributes of people were more highly favoured. Specifically, the sharing of real‐life stories and anecdotes was far more attractive and influential compared to the provision of educational content created by tertiary educated experts.

*Sure, I love following her because she's an accredited dietitian who shares credible content, but I mainly follow because she's so real and honest … she doesn't use filters or facetune to alter her face or body. When she has acne, she doesn't hide it in her videos. She doesn't edit out her cellulite and stretch marks either which makes me feel good about myself and my body*. (Participant 2)

Notably, participants were not motivated to follow someone based on the number of social media followers they possessed, given follower numbers did not determine a person's authenticity. In addition, most participants acknowledged that followers can be amassed in inauthentic ways including via buying ‘fake’ followers. Moreover, several participants voiced their distrust of people who possessed significantly large followings, associating this with increased disconnectedness and inauthenticity.

It was also identified that diversity, inclusivity, and transparency were important for authenticity. This involved more than declaring information sources and sharing unfiltered imagery, rather, it required a deeper level of transparency that involves an awareness of culture, experience, background, and representation. Participant 3 spoke about ‘scientific racism’ and warned: ‘If you're representing one type of people with the information you're sharing, then you're not doing a service to all of the people’.

Theme 2: Social media content must be engaging and easy to understand. All participants reported that accessing social media had become a part of their daily routine, with many acknowledging that it had become an obsession.

*…It's such a time suck … it's designed to pull you in. The scrolling is really easy, the algorithm is watching your every move so that recommendations are super accurate, it fuels the addiction to the content … it's a slippery slope*. (Participant 4)

Although participants viewed social media as a useful source of nutrition and health‐related information, this was not their primary reason for using it. Rather, participants generally accessed social media to ‘zone out’ from everyday life; try new recipes; learn about different cultures and their foods; communicate with friends and/or likeminded people; and stay abreast of current affairs.

Social media characteristics that resonated the most among participants included content featuring infographics, given they ‘*…highlight key points of information in fun and interesting ways*’ (Participant 5). In addition, most participants preferred captions that were short and simple, given ‘*…a very simple message can be incredibly effective*’ (Participant 6). Social media posts that simplified complex nutrition and health‐related information were also highly valued. Colourful content was preferred over black and white content, and while there was no clear preference among participants for vibrant colours or muted tones, most preferred content that displayed a consistent look and feel. Video content was favoured more so than static post content, namely because participants found videos to be more interesting and less arduous ways to assimilate information. In addition, most participants preferred to access videos with closed captions, given this allowed them to view content in public places where listening to audio was not appropriate.

Participants with extensive social media experience highlighted the importance of sharing content regularly, given ongoing engagement is favoured, thus, rewarded by social media algorithms.

*Posting consistently, several times a week, and encouraging your followers to like, comment on, share, and/or save your posts assists the algorithms to prioritise your content by pushing it to the top of your follower's feeds*. (Participant 7)

In addition, most participants reported that they regularly found new content of interest via searching for specific hashtags including ‘#recipes’.

Theme 3: Selling on social media may dilute authenticity, diminish trust, and turn participants away from social media content. Participants were deterred by direct selling, ‘tagging’ multiple brands, sharing affiliate links, featuring discount codes, and/or displaying sponsored ‘#ads’.

*I used to really enjoy following this fitness influencer, but as soon as she reached 100,000 followers, her ego ballooned, she stopped sharing free content … Everything now has a price, everything is a sales pitch with an affiliate link. I lost trust in anything she said, so I unfollowed her*. (Participant 8)

The selling of nutrition and health‐related products was a significant deterrent. Participants spoke negatively of content providers who used health content as a vehicle to sell products.

*There are so many people on social media who mention they've got this health problem that has been occurring – on Monday, they'll mention that it's been a detriment to their life. On Wednesday they'll say they've done some research, they think they found something that they might share with their followers, but they want to try it for a bit longer because they only want to share things that are, y'know, worth sharing because they really value the trust their followers have in them. Two days later, there they are saying: ‘Here are the products that work! Use my discount code, it's an affiliate link, but y'know, it really worked for me, it fixed my health problem’ … It's the same pattern every single time*. (Participant 9)

Selling and product endorsement made participants question the authenticity of content.

*You can't fault someone for talking about something and getting paid an exorbitant amount of money for it, it is a business after all, but, honestly, I'm not a big fan of the product endorsements and the way they've evolved on social media because you never really know what's genuine*. (Participant 4)

## 4 DISCUSSION

Social media presents dynamic, low‐cost opportunities for health promotion to increase young adult women's food and health literacy.^30^ The aim of this research was to use IPA^23^ to develop a robust understanding of how young adult women 18–35 years use social media in free living situations, including the type/s of nutrition and health‐related content they prefer to view and why. Three themes were identified. The first theme reflected participants' preference for credible social media content delivered by people who are genuine. The second theme captured characteristics that support the design and delivery of high‐quality nutrition and health‐related content that gets noticed within the crowded social media space. The third theme encapsulated young adult women's distrust of product endorsements, especially the selling of nutrition and health‐related products via social media.

The strongest finding from this research was that young adult women seek social media content created by ‘real’ people who are not afraid to be themselves. Conversely, participants disliked overly produced content created by highly polished people who portray unrealistic lifestyles. In addition, while participants indicated their preference for credible, evidence‐based nutrition and health‐related information shared by qualified health professionals such as Accredited Practising Dietitians, they also turned to social media for entertainment, escape, inspiration, and connection with others. These findings are congruent with survey research commissioned by Instagram involving 21 000 respondents (13–64 years) from 13 countries who indicated their preference for entertaining (55%), authentic (53%), creative (53%) and informative (51%) social media content.^31^ Moreover, just 36% of respondents indicated a preference for beautifully produced content, whist only 27% indicated a preference for content endorsed by popular social media influencers.^31^

A number of sources highlight the importance of authenticity and trust when health professionals engage in social media.^19^, ^21^, ^32^ Some authors warn that blurred boundaries between personal and professional use of social media may cause loss of trust from clients, thus, recommend a separation of personal and professional activity where possible.^21^, ^33^, ^34^ Other authors advocate that health professionals should not be afraid to be themselves on social media, given the only way to create meaningful relationships is to be genuine.^19^, ^32^ Ultimately, dietitians need to find a balance between maintaining professional standards while being themselves, and sharing their authentic voice.^19^, ^32^

To foster authentic connection, it is recommended that dietitians share original content created themselves, using their unique voice. While outsourcing content creation to a social media agency may seem like a professional and time‐saving tactic, it risks misalignment of vision, goals, and personality that are central to authenticity.^35^ Content focusing on the practical implementation of health advice is more likely to appeal than content that is overtly educational. For example, sharing health‐supportive cooking tips and evidence‐based health ‘hacks’^36^ are good options for drawing attention to nutrition and health‐related education. To support increased visibility of content, dietitians may consider using hashtags, given they extend content reach beyond existing followers by allowing content to be categorised, thus, discoverable via in‐app searches. Any word or phrase with a ‘#’ placed in front of it can be turned into a hashtag, for example, ‘#nutrition’. Dietitians may also consider connecting regularly with their audience, for example, via Instagram Live^37^ to discuss trending health topics with respected colleagues who also possess a social media presence. A high amount of incorrect and potentially dangerous nutrition and health‐related misinformation is perpetuated via social media by non‐health professionals.^12^, ^13^, ^14^, ^15^, ^38^, ^39^ Thus, it is recommended dietitians share regular ‘myth busting’ posts and/or videos that identify and correct false or misleading health information, given their ethical obligation to do so.^40^ In addition, to attenuate existing racial, social, and gender‐related disparities, content shared via social media must remain inclusive of all race, ethnicities, socioeconomic status and genders.^41^ Inclusivity is crucial to authenticity, given it supports a reduction of existing barriers to health care and information created by ignorance, prejudice and misunderstanding.^42^

Participants in this study were sceptical of social media content connected with endorsing and/or selling nutrition and health‐related products. This presents an interesting conundrum, given endorsement and/or selling of evidence‐based health‐supportive products is arguably necessary for some private practice dietitians trying to earn a living. Thus, they are encouraged to consider the cost versus benefits of selling and/or endorsing products via social media. In addition, dietitians are recommended to seek out advice from their governing body/bodies and regulatory authorities to ensure they do not breach professional codes of conduct and laws pertaining to social media use. For example, Australian dietitians should consult the Therapeutic Goods Advertising Code 2021^43^ to ensure adherence to legislative requirements when/if posting about therapeutic goods that implicate public health interests. Furthermore, Dietitians Australia provides guidelines applicable to all health professionals considering endorsement and/or selling of products including books and/or supplements.^44^ These include avoiding endorsement and/or selling of products that: are part of a ‘pyramid selling’ enterprise and/or multi‐level marketing scheme; do not possess a strong evidence base; and are outside of the health professional's scope of practice.^44^

Participants in this study used social media for many reasons. Dietitians looking to use it for health promotion need to understand how to craft high‐quality content that gains and sustains young adult women's attention. Specific characteristics that appealed to participants the most included use of colour, as well as accounts that possess a consistent look and feel. Information shared via static posts as well as videos were desirable, however, video content must be captioned to ensure users can view and understand content without using audio in public places. Participants viewed the use of filters and/or applications that alter facial features and body shape as inauthentic, thus, they should be avoided. Graphical depictions of information were preferred. Given health literacy is an important determinant of health,^45^ all social media content should remain jargon‐free, thus, easy to understand among people with low individual health literacy.^46^ Social media training is also recommended to ensure best practice, expert use of the social media app/s chosen.

In conclusion, this study provides good insight into the social media practices and preferences of young adult women aged 18–35 years. Participants did interact with nutrition and health‐related content on social media. In addition, many indicated their preference to learn more about nutrition and health via social media content shared by qualified health professionals including Accredited Practising Dietitians. The provision of such information via social media is more likely to have impact if delivered in authentic, entertaining and well‐produced ways, given this will support content to stand out in a crowded space.

There is currently a lack of academic research about young adult women's free living social media practices and preferences. This small, qualitative investigation provides insight into the views and practices of women aged 18–35 y who regularly use social media. As with all qualitative investigations, this research provides deep information about a small number of individuals. Additional and different research is needed to see if the views of participants in this study match broader societal views. This study was implemented according to best practice in qualitative research,^24^ however, it must be acknowledged that the primary researcher was a novice interviewer. In hindsight, some questions would have been modified to prompt deeper consideration of issues such as contributors to authenticity. In addition, three participants possessed marketing/communication/social media expertise; two were student dietitians; and one was a public health nutritionist. Given their heightened interest in social media and/or health promotion, this may have impacted results. With that said, information provided by these participants adds rich insight, thus further value to the results and discussion presented.

The findings from this study can be used to inform dietitians how to attract young adult women to their social media content. More research is needed to assess whether nutrition and health‐related content accessed via social media is effective at changing young adult women's nutrition and health‐related behaviours. In addition, it would be interesting for future research to investigate different age ranges and genders to assess whether social media is used differently, or if use changes over time, as people age.

## AUTHOR CONTRIBUTIONS

MM and DS planned the research. DS (the primary researcher) collected and analysed all data with support from MM and CK‐A. DS prepared the final manuscript with support from MM and CK‐A.

## CONFLICT OF INTEREST

The authors declare no conflicts of interest.

## Supporting information


**Data S1** Line of questioning for semi‐structured interviews with young adult women

## Data Availability

Research data are not shared.
