# Supplementary material for: #Healthpromotion: A qualitative exploration of how dietitians can use social media to positively influence women aged 18–35 years
Source: Nutr Diet. 2022 Sep 9;79(4):489–96. doi: 10.1111/1747-0080.12765 (PMC9546398; doi:10.1111/1747-0080.12765)
Supplement: Supplementary file 1 — Data S1 Line of questioning for semi‐structured interviews with young adult women [file NDI-79-489-s001.docx]

**Line of Questioning for Semi-structured Interviews with Young Adult Women.**

Note: Depending on participant responses to each question, probes are also provided to seek clarification and extend line of questioning.

**In your own words, can you tell me what you think about social media?**

**Can you tell me generally about your social media use? - how many years you’ve been using social media? How many times per week? How many hours per day?**

**What types of information do you like to access/view on social media?**

**Can you tell me about the social media app (or apps) you use and what you like about them?**

**Can you tell me what types of content you look for and what you like on social media?**

**Do you use social media to access nutrition and/or health related information?**

Probe: do you access information that helps you decide what to eat and/or when to eat?

Probe: do you access health about conditions or illnesses?

**What kinds of accounts and/or people (‘social media influencers’) do you follow on social media?**

Probe: what about that account and/or person do you like?

Probe if diet or health related: is the person qualified?

Probe: do you access information from dietitians?

Probe: do you access information from nutritionists?

Probe: do you access information from other health professionals, for example, doctors?

Probe: how do you know if you’re accessing information that is correct?

**Who are some of your favourite social media influencers and/or social media accounts?**

Probe: how long you’ve followed them?

Probe: what drew you to follow them?

Probe: is there anything that makes these accounts more engaging than others?

Probe: do any of these people and/or accounts share nutrition and/or health information?

Probe: has any of this information influenced your daily diet and/or health practices?

**There are social media accounts and influencers who endorse and/or sell diet and/or health related products, for example, supplements, protein powders, and recipe e-books. Have you ever purchased any of these items via social media?**

Probe: was this a once-off purchase?

Probe: was it helpful?

Probe: how did you determine that it was helpful?

Probe: what do you think about people who sell nutrition and health related products?

**How do you determine whether to trust information that you see on social media?**

**How do you decide whether or not to follow someone on social media?**

**Does someone’s follower number influence that way you think about the account and/or the person?**

Probe: what do you think about someone with a large social media following?

Probe: what do you think about someone with a small social media following?

**When using social media, do you access static posts or videos, or both?**

**Are there specific visual aspects of social media posts and/or videos that you find most engaging?**

Probe: what do you think about colourful social media posts?

Probe: are there specific colours or tones that you prefer?

Probe: what do you think about black and white social media posts?

Probe: what do you think about graphic depictions of information on social media?

Probe: do you read information on social media posts?

Probe: what do you think about short posts?

Probe: what do you think about long posts?

**Are there specific audio aspects of social media posts and/or videos that you find most engaging?**

Probe: what do you think about stories, reels, and lives?

Probe: are there any aspects about stories, reels, and lives that you prefer?

**Do you have any further thoughts or information that you would like to share about social media?**
